# Methylation-Dependent Binding of the Epstein-Barr Virus BZLF1 Protein to Viral Promoters

**DOI:** 10.1371/journal.ppat.1000356

**Published:** 2009-03-27

**Authors:** Sarah J. Dickerson, Yongna Xing, Amanda R. Robinson, William T. Seaman, Henri Gruffat, Shannon C. Kenney

**Affiliations:** 1 McArdle Laboratory, Departments of Oncology and Medicine, University of Wisconsin School of Medicine and Public Health, Madison, Wisconsin, United States of America; 2 Lineberger Comprehensive Cancer Center, University of North Carolina School of Medicine, Chapel Hill, North Carolina, United States of America; 3 Laboratoire de Virologie U758, ENS-Lyon, INSERM, Lyon, France; Emory University, United States of America

## Abstract

The switch between latent and lytic Epstein-Barr virus (EBV) infection is mediated by the viral immediate-early (IE) protein, BZLF1 (Z). Z, a homologue of c-jun that binds to AP1-like motifs (ZREs), induces expression of the BRLF1 (R) and BRRF1 (Na) viral proteins, which cooperatively activate transcription of the Z promoter and thereby establish a positive autoregulatory loop. A unique feature of Z is its ability to preferentially bind to, and activate, the methylated form of the BRLF1 promoter (Rp). To date, however, Rp is the only EBV promoter known to be regulated in this unusual manner. We now demonstrate that the promoter driving transcription of the early BRRF1 gene (Nap) has two CpG-containing ZREs (A**C**GCTCA and T**C**GCC**C**G) that are only bound by Z in the methylated state. Both Nap ZREs are highly methylated in cells with latent EBV infection. Z efficiently activates the methylated, but not unmethylated, form of Nap in reporter gene assays, and both ZREs are required. Z serine residue 186, which was previously shown to be required for Z binding to methylated ZREs in Rp, but not for Z binding to the AP1 site, is required for Z binding to methylated Nap ZREs. The Z(S186A) mutant cannot activate methylated Nap in reporter gene assays and does not induce Na expression in cells with latent EBV infection. Molecular modeling studies of Z bound to the methylated Nap ZREs help to explain why methylation is required for Z binding, and the role of the Z Ser186 residue. Methylation-dependent Z binding to critical viral promoters may enhance lytic reactivation in latently infected cells, where the viral genome is heavily methylated. Conversely, since the incoming viral genome is initially unmethylated, methylation-dependent Z activation may also help the virus to establish latency following infection.

## Author Summary

In cells with long-term latent Epstein-Barr virus (EBV) infection, the majority of the EBV genome becomes highly methylated. Methylation of cytosines plays a critical role in inhibiting the expression of cellular genes. In contrast, our laboratory previously showed that the EBV protein, BZLF1 (Z), which mediates viral reactivation and replication, preferentially binds to, and activates, the methylated form of the viral BRLF1 promoter. To date, however, BRLF1 is the only EBV promoter known to be activated by Z in this unusual manner. Here, we show that another EBV promoter (Nap, driving transcription of the *BRRF1* gene) likewise has two methylation-dependent Z binding sites, and that Z only activates the Nap efficiently in the methylated form. Molecular modeling studies suggest why methylation of the Nap enhances Z binding. Since the *BRLF1* and *BRRF1* genes encode essential viral transcription factors that work cooperatively with Z to induce the lytic form of viral infection, our results indicate that methylation of the EBV genome enhances Z-mediated disruption of viral latency.

## Introduction

Epstein-Barr virus (EBV) is a human gammaherpesvirus associated with B-cell lymphomas, nasopharyngeal carcinoma (NPC) and gastric cancer [1],[2]. EBV primarily infects two cell types, epithelial cells and B cells [1],[3]. As is the case for all herpesviruses, EBV can infect cells in either latent, or lytic, forms. Lytic replication, which is required for horizontal spread of the virus from cell to cell, and from host to host, occurs in epithelial cells and following differentiation of B cells into plasma cells [1], [4]–[7]. Lytic replication is mediated by the virally encoded DNA polymerase using the oriLyt replication origin, and results in the release of infectious viral particles [8]. In contrast, during latent viral infection (which normally occurs in memory B cells), only a subset of viral genes is expressed, the genome is replicated once per cell cycle using the cellular DNA polymerase and the oriP replication origin, and progeny virus is not released. Latent EBV infection allows the virus to persist for the life of the host and avoid detection by the immune system [1],[4]. Thus, both the latent and lytic forms of EBV infection are essential for viral pathogenesis.

The switch from latent to lytic infection is mediated by the immediate-early (IE) proteins BZLF1 (Z) and BRLF1 (R) [4], [6], [9]–[11]. Z and R are transcription factors which activate one another's promoters, as well as their own promoters [8]. In combination, Z and R induce expression of all early lytic viral proteins, allowing the virus to replicate. Z is a bZip protein homologous to c-jun and c-fos that binds to the consensus AP1 motif as well as atypical AP1-like motifs known as Z-responsive elements (ZREs) [12]–[15]. R activates some early promoters through a direct binding mechanism, but activates the BZLF1 promoter indirectly through effects on cellular transcription factors (c-jun and ATF-2) binding to a CRE motif [16],[17]. An early viral protein, Na (encoded by the *BRRF1* gene located on the opposite strand of the first R intron), induces c-jun phosphorylation and cooperates with R to enhance *BZLF1* transcription [18],[19]. The Na homologues in KSHV (ORF49) and MHV-68 (ORF49) likewise collaborate with their R homologues (KSHV ORF50 and MHV-68 Rta) to activate lytic viral promoters [20],[21].

The EBV genome is not methylated in virions. However, in cells with long-term latent infection, the majority of the EBV genome becomes highly methylated [22]–[24]. DNA methylation, which plays a critical role in modulating the expression of both cellular and viral genes, induces transcriptional repression by multiple different mechanisms, including prevention of transcription factor binding to DNA and the recruitment of HDAC complexes [25]–[33]. Surprisingly, while DNA methylation of the EBV IE *BRLF1* promoter (Rp) inhibits its activation by cellular transcription factors, it enhances the ability of Z to activate the Rp [34]. This unusual effect of Rp methylation on Z activation is due to the enhanced ability of Z to bind to the methylated, versus unmethylated, forms of two atypical CpG-containing Rp ZRE sites (Rp ZRE2 and Rp ZRE3), and requires serine residue 186 in the basic DNA domain of Z [22],[34]. The Rp also has one CpG-free ZRE, and can be activated by Z in the unmethylated form, albeit it less efficiently [34]. Although the crystal structure of Z bound to the consensus AP1 site has been previously published [35], the structure of Z bound to methylated site has not been published.

Z is the only transcription factor known to preferentially activate the methylated form of a target promoter. To date, however, the Rp is the only EBV promoter shown to have CpG-containing ZREs. A variety of studies have suggested that Rp activation is the essential first step required for Z disruption of viral latency, and that both Z and R expression are required for induction of most early lytic genes in the context of the intact viral genome [36],[37]. The apparently unique effect of methylation on Z binding to the Rp, but not other lytic viral promoters, may ensure that at the earliest stages of viral reactivation limiting amounts of Z are initially bound to the Rp rather than other lytic viral promoters.

In this paper, we demonstrate that the Na early lytic viral promoter (Nap) has two CpG-containing ZREs that can only be bound by Z in the methylated form. Furthermore, we show that Nap methylation is required for efficient Z activation of the Nap. We also demonstrate that the CpG motifs in the Nap ZREs are usually methylated on the EBV genome during latent infection, and that a Z mutant, Z(S186A), which cannot bind to the methylated Nap ZREs *in vitro* is unable to activate Na expression in latently infected cells. Our molecular modeling of Z bound to the newly discovered methylated ZREs in the Nap, versus the consensus AP1 site, helps to explain why methylation is required for Z binding to some ZREs, and the role that the Z Ser186 residue plays in this binding. The apparently unique ability of Z to activate the methylated forms of the R and Na promoters, but not other lytic EBV promoters, may be a mechanism by which the virus ensures that R and Na are the first genes activated during Z-mediated reactivation in latently infected cells. Conversely, the inability of Z to activate R and Na transcription efficiently from the incoming unmethylated viral genome may serve to promote viral latency.

## Materials and Methods

### Cell lines

293 cells infected with the Z-KO virus (293 Z-KO) were a gift from Henri-Jacques Delecluse and have been described previously [38]. 293 Z-KO were maintained in Dulbecco's modified Eagle medium (DMEM) containing 10% fetal bovine serum (FBS), 1% penicillin-streptomycin, and hygromycin B (100 µg/mL; Roche). DG75, an EBV-negative B-cell lymphoma line, Raji, an EBV-positive Burkitt lymphoma cell line, HONE-1 (a gift from Ronald Glaser), an EBV-negative human nasopharyngeal carcinoma (NPC) cell line, and SNU-719, a naturally derived EBV-infected gastric carcinoma cell line [39], were maintained in RPMI 1640 medium with 10% FBS and 1% penicillin-streptomycin. HeLa, a cervical adenocarcinoma line, were maintained in DMEM with 10% FBS and penicillin-streptomycin. HaCaT, a human keratinocyte cell line [40], were maintained in F media with 10% FBS and penicillin-streptomycin. HONE-1/EBV (a gift from Lawrence Young) is a human NPC cell line that stably maintains the EBV (Akata strain) genome under G418 selection in a latent form [41]. HONE-1/EBV cells were maintained in RPMI medium 1640 with 10% FBS, penicillin-streptomycin and G418 (400 µg/mL; Sigma).

### Plasmids

Plasmid DNA was purified through columns as described by the manufacturer (QIAGEN). Nap-LUC contains the Nap sequences from +76 to −209 (relative to the *BRRF1* transcription start site) inserted upstream of the luciferase gene in the pGL3-basic vector (Promega). Mutations to the Nap-LUC construct were created using the QuikChange site-directed mutagenesis kit (Stratagene). Rp-LUC contains the Rp sequences from +37 to −1069 (relative to the *BRLF1* transcription start site) inserted upstream of the luciferase gene in the pGL3-basic vector (Promega). BHLF1p-LUC was constructed by PCR amplifying the divergent BHLF1 and BHRF1 promoter sequences (from 52781 to 53797) within the oriLyt region from EBV B95.8 genomic DNA and inserting it in the pBSLUCCAT construct; this plasmid contains the firefly luciferase gene under the transcriptional control of the BHLF1 promoter and CAT gene under the control of the BHRF1 promoter. The Z expression vector (pSG5-Z) contains genomic Z downstream of the SV40 promoter (a gift from S. Diane Hayward) in the pSG5 vector (Stratagene). The Z cDNA (a gift from Paul Farrell) was cloned into the pSG5 vector to create pSG5-ZcDNA, which was used to *in vitro* translate the Z protein. Z(S186A) and Z(C189S) mutations in the pSG5-ZcDNA vector were constructed using the QuikChange site-directed mutagenesis kit (Stratagene) to allow *in vitro* translation of the mutant proteins. The R expression plasmid contains genomic R sequences downstream of the SV40 promoter in the pSG5 vector (a gift from S. Diane Hayward).

### DNA transfection

Transfections of 293 Z-KO and HONE-1 cells were performed by use of Lipofectamine 2000 (Invitrogen). Cells (8×10^5^) were seeded the day prior to transfection in 2 mL of medium in a six-well plate. Transfections were performed according to the manufacturer's instructions, except that the reagent∶DNA ratio was 3 µL∶1.5 µg. Transfections of HeLa and HaCaT cells for reporter assays were performed by use of Fugene 6 (Roche) according to the manufacturer's instructions. DG75 cells were electroporated using 10 µg DNA with the Bio-Rad Gene Pulser Xcell device, using 250 V, 975 µF, ∞ Ω, 4 mm cuvettes.

### Immunoblotting

Immunoblotting was performed as described previously [22],[42]. Briefly, cells were lysed in SUMO lysis buffer; equivalent amounts of protein were then separated in sodium dodecyl sulfate-10% polyacrylamide gel electrophoresis gels (SDS-PAGE) and transferred to membranes. Membranes blocked in 5% milk then incubated at room temperature for 1 h with the appropriate primary antibodies diluted in 5% milk in 1× PBS and 0.1% Tween 20 (PBS-T). Primary antibody dilutions were as follows: 1∶250 anti-BRLF1 (Argene), 1∶250 anti-ZEBRA (Santa Cruz, sc-53904), 1∶250 anti-EA-D (Vector), 1∶300 anti-Na rabbit antibody [18] and 1∶5,000 anti-β-actin (Sigma). After washing, the appropriate horseradish peroxidase conjugated secondary antibodies (Pierce) were used at a dilution of 1∶7,000 in 5% milk in 1× PBS-T for 1 h at room temperature and washed. Bound antibodies were visualized by use of enhanced chemiluminescent reagent (Pierce) according to the manufacturer's instructions.

### *In vitro* DNA methylation

*In vitro* DNA methylation of the luciferase constructs was accomplished with CpG methylase (*SssI methyltransferase*; New England Biolabs), by following the procedure recommended by the manufacturer. Completion of DNA methylation was confirmed by digestion with the restriction enzyme *Hpa*II (New England Biolabs), which cleaves its recognition sequence only if the DNA is not methylated at the cytosine residue within the CpG motif.

### Luciferase assays

Luciferase assays were performed 48 h after transfection by using extracts prepared by freeze-thawing the cell pellet in reporter lysis buffer according to the instructions of the manufacturer (Promega). Luciferase activity was assayed using the luciferase reporter assay system (Promega) as suggested by the manufacturer.

### Chromatin immunoprecipitation (ChIP) assay

293 Z-KO cells (3×10^7^ cells per condition) were transfected with a control vector or vector encoding Z and harvested after 20 h for ChIP assays using a modified Upstate ChIP protocol (Millipore, Billerica, MA). Chromatin was sonicated to ∼400 bp. Antibodies used were control goat polyclonal immunoglobulin G (IgG)(Santa Cruz, sc-2028) and goat anti-ZEBRA (vE-20) (Santa Cruz, sc-17503). Input (total) DNA was obtained from samples not incubated with antibody. ChIP DNA was analyzed using the following primers: Nap (5′-CCC TGT TGT TTC GGA GAA TGG CCC-3′ and 5′-GGA AGA CTT TCT GAG GCT AAC TCC TG-3′); oriLyt (5′-GCG TCT GGA CGA CGC TGG CGA-3′ and 5′-CTC CAG GTA CCA CCC ACC TGG TG-3′) and BALF5 (5′-GCG TAG AAG TAG GCC TGC T-3′ and 5′-CGG AAG CCC TCT GGA CTT C).

### Probes for electrophoretic mobility shift assay (EMSA)

A series of probes spanning the BRLF1 promoter sequences (from −1 to −270) or the BRRF1 (Nap) promoter sequences from −280 to −470, −108 to −301, and +77 to −128 (relative to the BRRF1 mRNA start site) were created by PCR amplification and were ^32^P end-labeled with T4 Polynucleotide Kinase (New England Biolabs) in accordance with the manufacturer's instructions. In addition, a series of oligonucleotide probes were synthesized as shown below (with positions of the oligonucleotides in the EBV genome relative to the *BRRF1* start site shown in parentheses). ZRE sequences are underlined. The oligonucleotides were synthesized without methylated cytosines at the CpG motif(s) within potential ZRE sites (MWG Biotech). The double-stranded oligonucleotides were either left unmethylated or methylated *in vitro* using *M. Sss*I CpG methylase (New England Biolabs), then purified by phenol-chloroform extraction and ethanol precipitation. Oligonucleotides containing Rp ZRE2, Rp ZRE3 and a consensus AP1 site from the EBV *BMRF1* promoter were also synthesized. Additionally, a series of oligonucleotides with or without methylated cytosines at the CpG motif(s) in potential Nap ZREs were synthetically synthesized (Sigma). Double-stranded oligonucleotide probes were end labeled with ^32^P using T4 Polynucleotide Kinase. Oligonucleotide sequences are as follows (with the cytosine of the CpG motif is shown in boldface type): Nap ZRE-1 (−41 to −58) 5′-CAT TCT **C**GC C**C**G TGG GCC-3′ and 5′-GGC CCA **C** GG G**C**G AGA ATG-3′; Nap ZRE-2 (−70 to −83) 5′-GTT GAG **C**GT GGC CA-3′ and 5′-TGG CCA **C**GC TCA AC-3′, BMRF1 AP1 5′-GAT GAC CTT TGA GTC AGG TGG CTA-3′ and 5′-TAG CCA CCT GAC TCA AGG GTC ATC-3′, Rp ZRE3 5′-TAT AGC AT**C** G**C**G AAT TTT-3′ and 5′-AAA ATT **C**G**C** GAT GCT ATA-3′, Rp ZRE2 5′-TAA AAT **C**GC TCA TAA GCT TA-3′ and 5′-TAA GCT TAT GAG **C**GA TTT TA-3′.

### EMSAs

*In vitro* translated wild-type and mutant Z proteins were generated using TNT T7 Quick Coupled Transcription/Translation System (Promega) in accordance with the manufacturer's instructions. Z binding reactions were carried out in a buffer consisting of 100 mM KCl, 20 mM HEPES (pH 7.3), 10% glycerol, 0.2 mM EDTA and 4 mM dithiothreitol (DTT) with 2 µg of poly(dI/dC)/poly(dI/dC) (Pharmacia). 2 µL of *in vitro* translated protein were added to each reaction and incubated at 4°C for 10 min before adding labeled probe (20,000 c.p.m.). The reactions were incubated for 30 min at 4°C before being loaded onto a 4% polyacrylamide gel and separated in 0.5× Tris-borate-EDTA buffer at 35 mA at room temperature. Image quantifications were performed using ImageQuant software.

### Determining the methylation status of Nap in the viral genome of EBV-infected cell lines

Total genomic DNA was isolated from cultured EBV-positive cells using the DNeasy tissue kit (QIAGEN). Bisulfite modification of genomic DNA was performed using the EpiTect bisulfite kit (QIAGEN) in accordance with the manufacturer's instructions. The modified DNA was amplified by PCR using the EBV *BRRF1* promoter primers: 5′-GTGTTTATGGTGGTAGGAATTATTAT-3′ and 5′-CAACTAAACTCTCTAATCTCTAACTAC-3′. Thermal cycler conditions for the PCR were as follows: 1 cycle at 95°C for 10 m followed by 35 cycles of 95°C for 30 s, 52°C for 40 s, 70°C for 40 s, and a final extension of 72°C for 7 m. PCR products were agarose gel purified with a QIAGEN gel extraction kit and then cloned into the pT7Blue Perfectly Blunt Cloning Kit (Novagen). For each cell line, 5 to 10 clones were sequenced to determine the methylation status of the Na promoter.

### Molecular modeling

Structures of Z bound to four different ZREs were modeled based on the crystal structure of Z (S186A, C189S) bound to the AP1 site (PDB code: 2c9l) using the Sybyl program. Residues 186 and 189 of Z were changed back to wild type sequence; and the AP1 sequence was replaced by the sequence of each ZRE followed by addition of a methyl group to the C5 position of each CpG. Modeling was finished by several rounds of manual model building and energy minimization.

### Accession numbers

EBV genome, type 1 NC_007605; Z (BZLF1) Swiss-Prot P03206.

## Results

### Z preferentially binds to the methylated form of the Na promoter (Nap)

**Figure 1:**
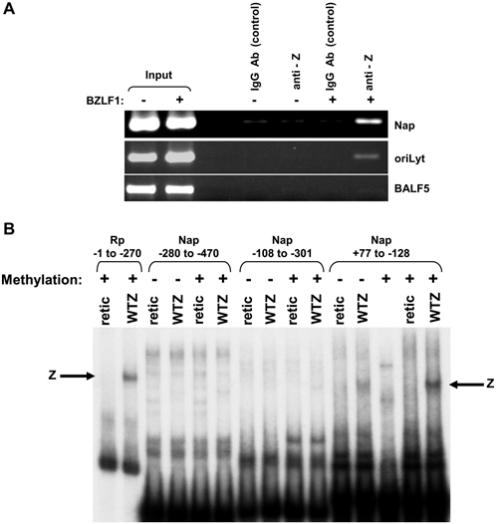
Z binds to the Na promoter, and binding is enhanced by Nap methylation. (A) Latently infected EBV+ 293 Z-KO cells were transfected with a BZLF1 expression vector (+) or an empty vector control (−). Chromatin immunoprecipitation assay was performed 20 hours after transfection using anti-Z and control goat IgG antibodies as indicated to examine Z binding to the Na promoter (Nap), ZREs within the EBV oriLyt (positive control) and EBV sequences in the 3′ end of the EBV BALF5 gene (negative control). (B) The ability of *in vitro* translated Z to bind to ^32^P end-labeled probes (in either the methylated, or unmethylated forms) containing the BRRF1 promoter (Nap) sequences from −280 to −470, −108 to −301, and +77 to −128 (relative to the *BRRF1* mRNA start site) was examined by EMSA. A probe containing the BRLF1 promoter (Rp) sequences from −1 to −270 (in the methylated form) served as a positive control.

Regulation of the Nap by viral and cellular factors has not been well studied. Although the Nap was previously reported to be activated by Z, but not R, in reporter gene assays, specific ZRE sites required for Z activation of the Nap were not defined [18]. Furthermore, although the Nap has a number of potential CpG-containing ZRE motifs, the effect of methylation on Z binding to the Nap has not previously been examined. To determine if Z binds to the Nap in the context of the intact viral genome, latently EBV infected 293 cells were transfected with a Z expression vector or a control vector, and ChIP assays were performed 20 hours later to detect Z binding. As shown in Fig. 1A, the ChIP assays confirmed that Z binds to the Nap *in vivo*.

To locate potential ZRE sites in the Nap, we performed EMSAs using *in vitro* translated Z and a series of unmethylated or methylated probes spanning the Nap sequences between −470 and +77. The methylated form of a probe containing BRLF1 promoter (Rp) sequences (from −1 to −270) served as a positive control for Z binding. The only Nap probe that had detectable Z binding contained Nap promoter sequences from −128 and +77 (relative to the *BRRF1* transcriptional start site)(Fig. 1B). Furthermore, Z bound to the methylated form of this Nap probe more efficiently than to the unmethylated form. These results suggested that Z may preferentially activate the methylated form of Nap.

### Methylation of the Nap inhibits its constitutive activity

**Figure 2:**
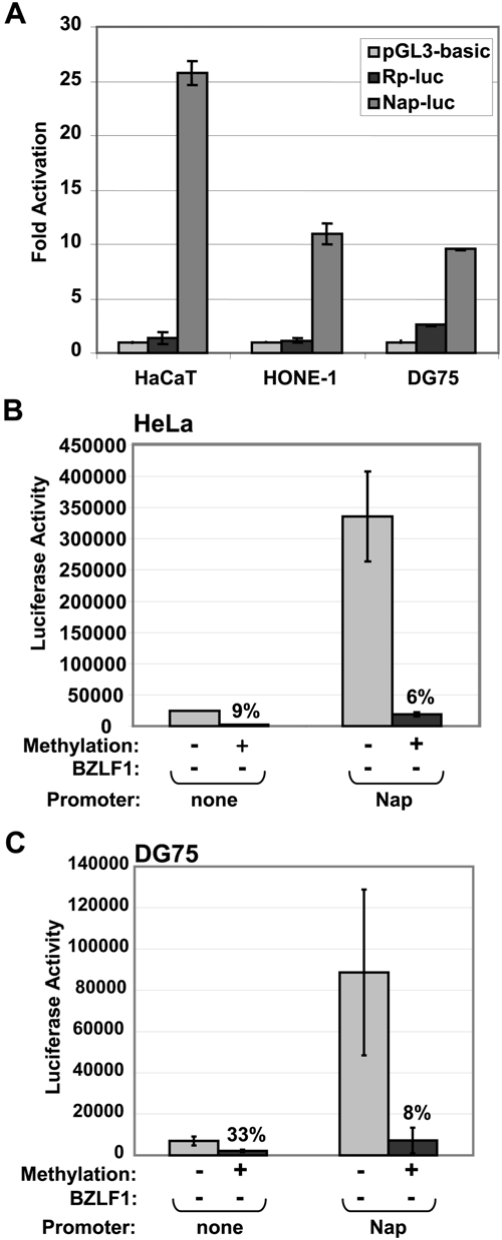
Methylation inhibits constitutive activity of the Nap. (A) Reporter constructs containing no promoter (pGL3-basic), the R promoter (Rp-LUC) or the Na promoter (Nap-LUC) driving luciferase were transfected into HONE-1, HaCaT or DG75 cells. The luciferase activity produced by each vector is shown (relative to the luciferase activity of the promoterless vector, set as 1). HeLa (B) and DG75 (C) cells were transfected with reporter constructs containing no promoter (none) or the Na promoter (Nap) driving luciferase. Luciferase vectors were either mock-methylated or methylated *in vitro* using *M.Sss*1 prior to transfection as indicted. The decrease in luciferase activity derived from the methylated versus unmethylated promoter constructs is indicated (activity of the unmethylated constructs is set at 100%).

Although promoter DNA methylation generally inhibits the ability of cellular transcription factors to activate gene expression, we previously showed that methylation of the EBV Rp enhances its ability to be activated by Z in co-transfection reporter gene assays [22],[34]. To examine how Nap methylation affects its ability to be activated by cellular and viral factors, we inserted the Nap sequence upstream of the luciferase gene to create the Nap-LUC construct. In the absence of promoter DNA methylation, Nap had strong constitutive activity in two different EBV-negative epithelial cell lines (HaCaT and HONE-1) as well as an EBV-negative B cell lymphoma line (DG75)(Fig. 2A). In comparison to the Nap, the constitutive activity of an Rp-driven luciferase construct (Rp-LUC) was much weaker in all three cell lines. These results suggest that the unmethylated form of the Nap has strong constitutive activity in a variety of EBV-negative cell lines.

To examine the effect of Nap methylation on its activation by cellular factors, we methylated or mock-methylated the Nap-LUC vector, or the promoterless luciferase vector (pGL3-basic), with *M.Sss*I prior to transfection. Digestion of the reporter constructs by *Hpa*II was used to verify their methylation status (data not shown). The constitutive activity of the methylated Nap-LUC vector was much lower than that of the unmethylated construct in both HeLa cells (Fig. 2B) and DG75 cells (Fig. 2C). The low level activity of the promoterless control vector (presumably driven by cryptic binding sites for cellular transcription factors) was likewise decreased by methylation. These results indicate that methylation of the Nap inhibits the ability of cellular transcription factors to activate this promoter.

### Methylation of the Nap is required for efficient Z activation

**Figure 3:**
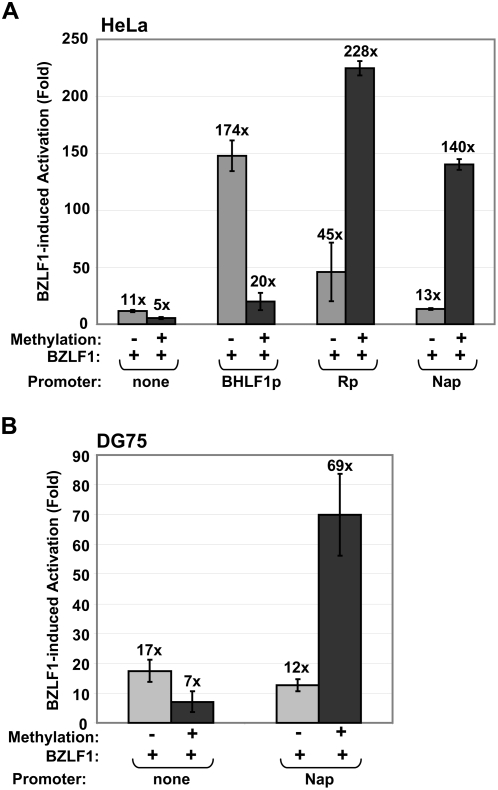
Methylation of the Nap promotes Z activation. HeLa (A) and DG75 (B) cells were transfected with a promoterless luciferase vector, or luciferase vectors driven by the Na, R, or BHLF1 lytic EBV promoters, in the presence or absence of a co-transfected Z expression vector. Luciferase vectors were either mock-methylated or methylated *in vitro* using *M.Sss*1 prior to transfection as indicted and the amount of luciferase activity determined 48 hours after transfection. The fold-increase in promoter activity in the presence of co-transfected Z (versus a vector control) is shown (obtained from duplicate transfections).

To examine how methylation of the Nap affects its ability to be activated by Z, we transfected HeLa cells with methylated or mock-methylated forms of the Nap-LUC vector in the presence or absence of a Z expression vector. We also examined the effect of methylation on Z activation of Rp-LUC, BHLF1p-LUC (a luciferase vector driven by the early viral BHLF1 promoter, in which the known ZREs cannot be methylated), or the promoterless control luciferase vector (pGL3-basic). Z clearly activated the methylated form of Nap-LUC much more efficiently than the unmethylated form (140-fold activation versus 13-fold) in HeLa cells (Fig. 3A). Similar results were obtained in the EBV-negative B cell line, DG75 (Fig. 3B). As previously described [34], methylation of the Rp also enhanced its ability to be activated by Z (Fig. 3A). In contrast to its effects on the Rp and Nap, methylation of the BHLF1p (which contains CpG-free ZREs) strongly inhibited its ability to be activated by Z (Fig. 3A). Z also induced detectable activation of the promoterless luciferase vector in HeLa and DG75 cells (perhaps reflecting the presence of a previously described cryptic AP1 motif in the luciferase vector) [43], and this activation was inhibited by methylation (Fig. 3A and 3B). These results indicate that both the Nap and Rp are preferentially activated by Z in the methylated form, consistent with the presence of methylation-dependent ZREs in their promoters.

### The Nap has two CpG-containing ZREs that are only bound by Z in the methylated form

**Figure 4:**
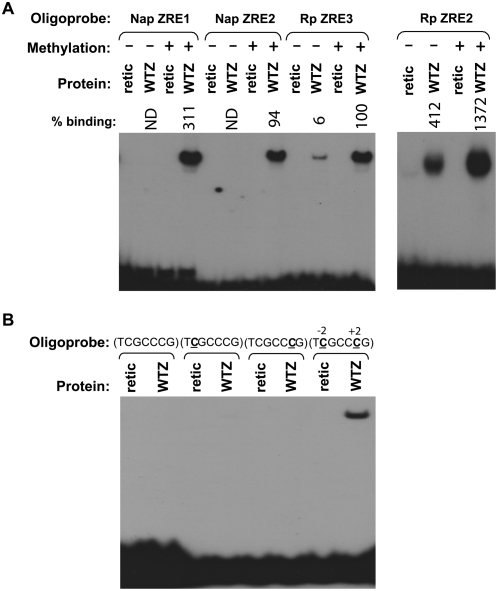
Z preferentially binds to the methylated forms of ZRE sites in the Nap. (A) The ability of *in vitro* translated Z to bind to labeled oligonucleotide probes containing potential ZRE sites in the Nap was examined by EMSA using methylated or mock-methylated probes as indicated. Nap sequences (with probable ZRE sites underlined) were (Nap ZRE1, −58 to −41): 5′-CAT TCT CGC CCG TGG GCC-3′ and (Nap ZRE2, −83 to −70): 5′-GTT GAG CGT GGC CA-3′. Binding to the unmethylated and methylated forms of two CpG-containing ZREs in the BRLF1 promoter (Rp) was also examined. The relative efficiency of Z binding to each probe is indicated; the amount of Z binding to the methylated Rp ZRE3 probe is arbitrarily set as 100%. ND: none detected. (B) The ability of *in vitro* translated Z to bind to the labeled oligonucleotide Nap ZRE1 probe (−58 to −41) containing no methylated cytosine, one methylated cytosine, or two methylated cytosines was compared. The methylated cytosine(s) present in each probe are indicated by a bold C in the sequence.

To map the precise location(s) of the ZREs in the Nap, we synthesized oligonucleotide probes spanning each of the various CpG motifs located within the Nap sequences between −128 and +77, as well as potential predicted ZREs without CpG motifs, and examined the ability of Z to bind to these probes in the methylated or unmethylated forms. We identified two CpG-containing ZREs, located between −53 to −47 (“Nap ZRE1”) and −81 to −75 (“Nap ZRE2”). Surprisingly, neither of these Nap ZRE sites is detectably bound by Z in the unmethylated form (Fig. 4A). In contrast, the CpG-containing ZRE sites in the Rp bind to Z in both the methylated and unmethylated forms, albeit more much efficiently in the methylated form (Fig. 4A). Thus, Z binding to the CpG-containing ZREs in the Nap is even more methylation-dependent than Z binding to the CpG-containing ZREs in the Rp. We did not identify any strong Z binding sites using a series of oligonucleotide probes containing potential unmethylated ZREs in the Nap (data not shown).

The Nap ZRE1 site (T**C**GCC**C**G) contains two CpG motifs. To determine if Z binding to the Nap ZRE1 motif requires methylation of one, or both, cytosines, we synthesized probes that contained one methylated cytosine at either CpG motif, or had both CpGs methylated. As shown in Fig. 4B, Z only binds to the Nap ZRE1 site when both cytosines are methylated. Thus, methylated cytosines at positions −2 and +2 are both essential for Z binding to the methylated Nap ZRE1 site.

The Nap ZRE2 site (A**C**GCTCA) is similar to the Rp ZRE2 site (T**C**GCTCA). Although the Rp ZRE2 site and the Nap ZRE2 site differ only at position −3 (which is a thymine in the Rp site versus an adenine in the Nap site), Z binds to both the unmethylated and methylated forms of the Rp ZRE2 site, whereas it only binds to the methylated form of the Nap (ZRE2 site) (Fig. 4A). Thus, this one basepair alteration in the ZRE is sufficient to convert the ZRE to completely methylation-dependent Z binding. In contrast to the Nap ZRE2 site, the Nap ZRE1 site (T**C**GCC**C**G) is quite different from all previously reported ZREs and is unique in containing methylated cytosines located at both positions −2 and +2.

### Nap ZRE1 and ZRE2 are both required for Z activation of methylated Nap

**Figure 5:**
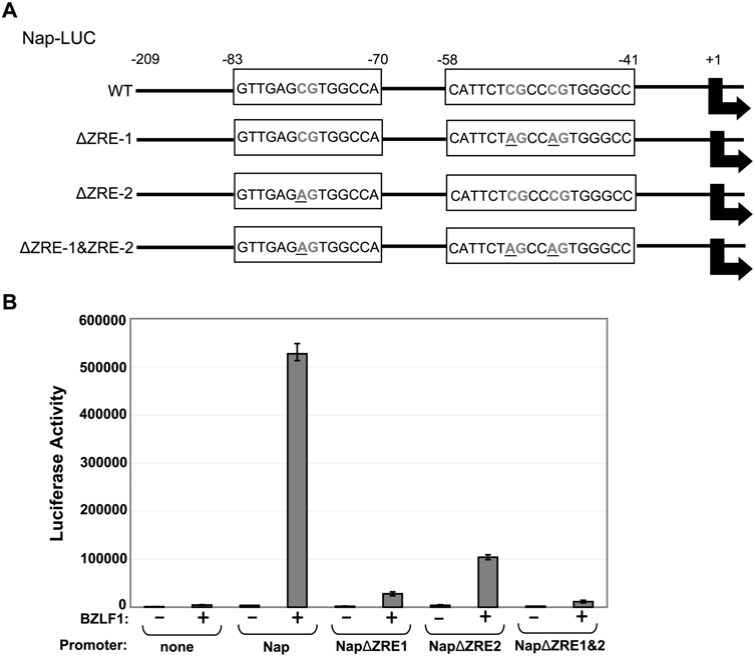
Nap ZRE1 and ZRE2 are required for Z activation of the methylated Nap. (A) site-directed mutations of the two ZREs in Nap-LUC were constructed as indicated. Specific base pairs altered are underlined. (B) HeLa cells were transfected with the methylated Nap-LUC constructs (wildtype versus mutant) in combination with either the empty vector (vector) or the Z expression vector (Z). The amount of luciferase activity determined 48 hours after transfection is shown (obtained from duplicate transfections).

To determine if both the Nap ZRE1 and ZRE2 sites are important for Z activation of the methylated Nap-LUC vector, we constructed site-directed mutants of the Nap-LUC that altered the ZRE1 and/or ZRE2 sites as shown in Fig. 5A. Z activation of methylated Nap-LUC was strongly reduced by mutation of either ZRE1 or ZRE2 (Fig. 5B). These results indicate that the ability of Z to activate the methylated form of Nap *in vivo* to a high level requires the presence of two different CpG-containing ZRE sites. Efficient Z activation of other early lytic viral promoters, such as BMRF1, has likewise been shown to require at least two ZREs [44],[45].

### Serine residue 186, but not cysteine residue 189, is required for Z binding to methylated Nap ZREs

**Figure 6:**
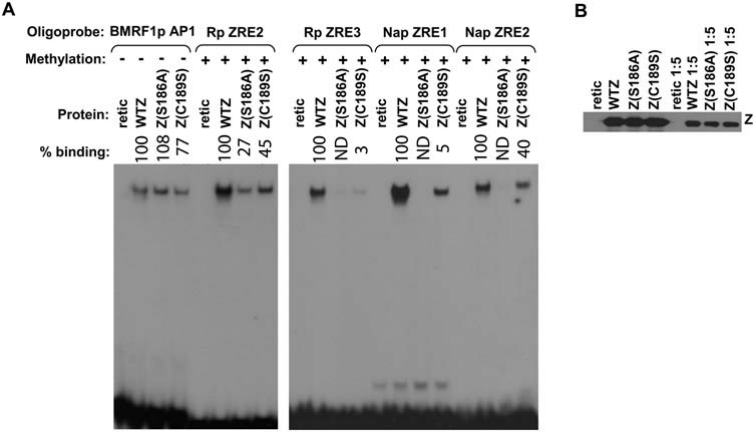
Serine residue 186 is required for Z binding to the ZREs in the Nap. (A) EMSA was performed using either methylated or mock-methylated oligonucleotide probes containing the Nap ZRE1, Nap ZRE2, Rp ZRE2, Rp ZRE3 and BMRF1p AP1 sites and *in vitro* translated wild-type Z (WTZ), ZS186A or ZC189S. The relative efficiency of WTZ, versus mutant Z, binding to each probe is indicated; the amount of WTZ binding to each probe is arbitrarily set as 100%. ND: none detected. (B) The amount of Z protein used in the EMSAs in (a) was quantitated by immunoblot for WT and mutant proteins.

A Z mutant in which the Ser186 residue in the basic DNA binding domain is converted to an alanine (the equivalent residue in c-jun and c-fos) binds poorly to the methylated forms of Rp ZRE2 and Rp ZRE3, but binds at least as well as wild-type Z to the consensus AP1 site [22]. More recently, converting Z residue Cys189 to a serine residue was reported to inhibit Z binding to methylated Rp ZRE3, but not AP1 [46]. To determine whether Z residue 186 or 189 regulates Z binding to methylated ZREs in Nap, we compared the ability of *in vitro*-translated wild-type Z, Z(S186A) and Z(C189S) to bind to labeled oligonucleotide probes containing the Nap ZRE1 and ZRE2 sites, the consensus AP1 site from the *BMRF1* early viral promoter, or the Rp ZRE2 and ZRE3 sites. The probes were synthesized such that they contained methylated cytosines at the CpG motifs. The Z(S186A) mutant was highly impaired in comparison to wild-type Z for binding to the methylated forms of the Nap ZRE1 and ZRE2 sites, as well as the Rp ZRE2 and ZRE3 sites, but, as expected, was able to bind to the consensus AP1 site at least as well as wild-type Z (Fig. 6A). The Z(C189S) mutant was slightly impaired for binding to the methylated forms of the Rp ZRE2 and Nap ZRE2 sites, but highly impaired for binding to the methylated forms of Nap ZRE1 and Rp ZRE3 (Fig. 6A). These results indicate that residue Ser186 is absolutely required for Z binding to three of the four known methylation-dependent ZREs (Rp ZRE3, Nap ZRE1 and Nap ZRE2), and greatly enhances Z binding to the other methylated ZRE (Rp ZRE2). In contrast, the requirement for Cys189 varies between the different methylation-dependent ZREs.

### Serine residue 186 is required for activation of the methylated Nap

**Figure 7:**
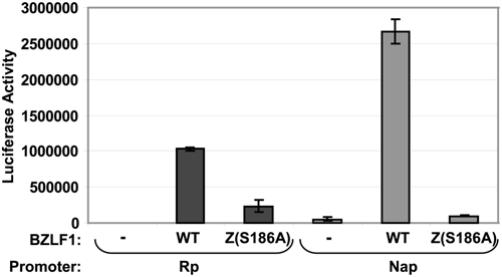
Serine residue 186 is required for Z activation of the methylated Nap *in vivo*. HeLa cells were transfected with methylated luciferase vectors driven by the Na or R promoters, in the presence or absence of a co-transfected wild-type Z (WTZ), or the Z(S186A) mutant altered in the DNA binding domain at residue 186. The amount of luciferase activity produced by each condition (obtained from duplicate transfections) is indicated.

To determine whether Z residue Ser186 is required for activation of the methylated Nap *in vivo*, we compared the ability of wild-type Z, versus the Z(S186A) mutant, to activate the methylated Nap-LUC construct. The methylated forms of the Nap-LUC or Rp-LUC vectors were co-transfected into HeLa cells with a control vector or vectors expressing either wild-type Z or Z(S186A). Consistent with its inability to bind to methylated Nap ZRE1 and ZRE2 in the EMSAs, Z(S186A) was impaired in the ability to activate the methylated form of Nap (Fig. 7). As previously reported [22],[34], Z(S186A) was also defective in activating the methylated form of Rp. These results indicate that Z Ser186 is important for activating the methylated forms of both Rp and Nap *in vivo*. Thus, the previously reported finding that the Z(S186A) mutant is unable to disrupt viral latency in the context of the intact viral genome [36],[37] may reflect not only its inability to activate the Rp, but also its inability to activate the Nap.

### The Nap is highly methylated in latently infected cells

Although the Rp was previously shown by our laboratory to be highly methylated in a variety of different latently infected EBV-positive cell lines [22], the methylation status of the Nap has not been previously reported. To examine this, we isolated DNA from a) 293 cells infected with a BZLF1-deleted virus, b) HONE-1/EBV cells (an NPC line super-infected with EBV), c) SNU-719 cells (a gastric carcinoma line which has remained EBV-positive in culture) or d) Raji cells (an EBV-positive Burkitt lymphoma line), and then treated the DNA with sodium bisulfite, converting unmethylated, but not methylated, cytosine residues into thymine residues. Following bisulfite treatment, the Nap sequence was PCR-amplified and cloned into the pT7Blue vector (Novagen). A number of different clones derived from each cell line were sequenced to determine the methylation status of the Nap. As shown in Table 1, the ZREs in the Nap were both highly methylated in each cell line. These results suggest that the ability of Z to bind to the methylated forms of the two ZREs in Nap is likely essential for its ability to activate Na transcription in latently infected cells.

**Table 1:** Methylation of the Nap ZREs in the EBV genome.

Cell Line	Description	ZRE2	ZRE1 −2	ZRE1 +2
**293-ZKO**	superinfected 293	5/5	5/5	4/5
**HONE-1/EBV**	superinfected NPC	7/8	7/8	7/8
**SNU-719**	EBV+ gastric	8/8	8/8	7/8
**Raji**	EBV+ BL	8/8	8/8	8/8

The cytosine methylation status of the CpG motifs within the ZRE1 and ZRE2 sites in the Nap in different latently infected EBV+ cell lines is shown. The number of clones found to contain a methylated CpG in each Nap ZRE, versus the number of clones sequenced in each cell line (following bisulfate treatment), is indicated.

### Wild-type Z but not Z(S186A) activates Na and R expression in latently infected 293 cells

**Figure 8:**
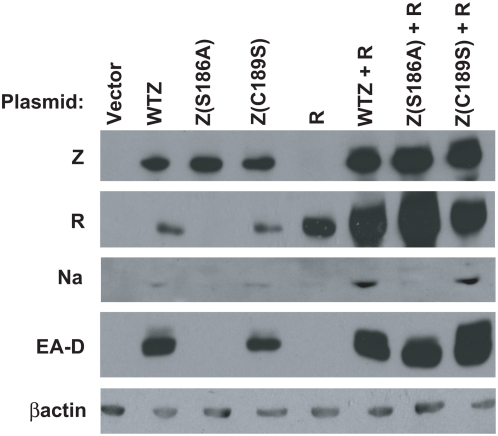
Wild-type Z, but not Z(S186A), activates Na and R transcription. Wild-type Z (WTZ), Z(S186A), Z(C189S) or empty vector (vector) were transfected in to 293 Z-KO cells in the presence or absence of an R expression vector and assayed by western blot for expression of Z, R, Na, EA-D or β-actin.

To determine if Z binding to the methylated form of the Nap is required for Z activation of Na expression in the context of the intact viral genome, we compared the ability of wild-type Z, the Z(S186A) mutant, or the Z(C189S) mutant to activate Na expression in the presence or absence of an R expression vector in 293 cells stably infected with a BZLF1-deleted mutant EBV. In the context of the intact latent viral genome, both Z and R are required to induce expression of most early lytic gene promoters [8]. As previously reported [36],[37], the Z(S186A) mutant by itself was not able to induce expression of the R protein, or the early viral protein EA-D (BMRF1) (Fig. 8). This defect was observed even when the Z(186A) mutant was expressed at extremely high levels (data not shown). However, in combination with an R expression vector, Z(S186A) activated EA-D, consistent with the known ability of this mutant to bind to the BMRF1 promoter ZREs. Furthermore, in comparison to wild-type Z, the Z(S186A) mutant was also impaired for the ability to activate expression of Na, and this defect persisted even when the R protein was supplied in *trans*. The Z(C189S) mutant was similar to wild-type Z in its ability to activate R, EA-D and Na expression, consistent with its ability to bind (at least weakly) to one or more ZREs in each promoter. These results confirm that the ability of Z to bind to the methylated form of the Nap is required for efficient Z activation of Na expression in the context of the intact viral genome in latently infected cells.

### Modeling Z interactions with methylated and unmethylated ZREs

**Figure 9:**
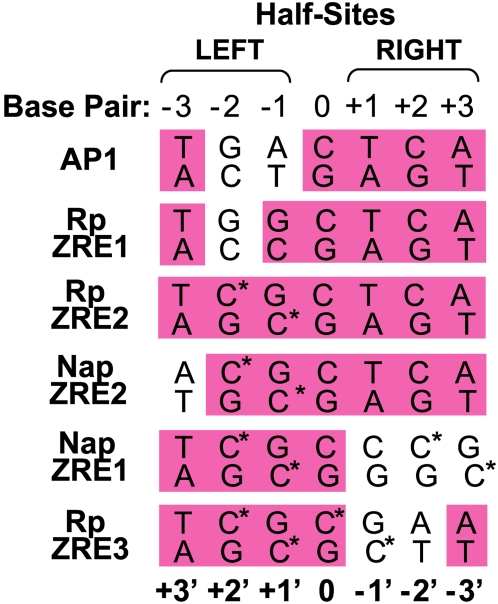
Comparison of ZRE sites. Sequences of the consensus AP1 site, the three Rp ZRE sites and the two Nap ZREs are shown. Methylated cytosines are indicated with an asterisk.

The sequences of the consensus AP1 site (from the BMRF1p), a ZRE which is bound by Z in the unmethylated form (from the Rp), and the four known methylation-sensitive ZREs (from the Rp and Nap) are compared in Fig. 9. Interestingly, the ZREs which are bound by Z in the methylated form all have a CpG motif in the left half-site at the same position (leading to methylated cytosines at −2 and +1').

**Figure 10:**
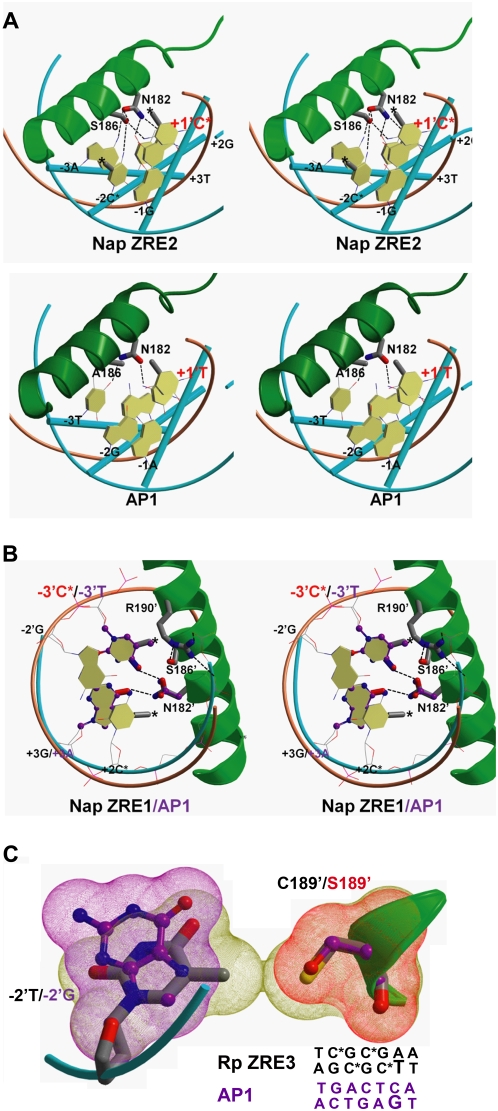
Modeling of Z binding to methylated ZRE sites versus AP1. (A) The predicted structure and hydrogen interactions between WT Z bound to Nap ZRE2 (upper panel, stereoview) is compared to Z(S186A) bound to the AP1 site (lower panel, stereoview). Z is shown as a green ribbon; hydrogen interactions are indicated by dotted black lines and the two DNA strands are shown as orange and cyan tubes. The left half-sites of Nap ZRE2 and AP1 are shown in cyan basepair stick as indicated. (B) Stereoview illustration of Z binding to the right half-site of methylated Nap ZRE1 is shown. A purple ball-and-stick model highlights the key difference of the Z/AP1 structure (at position −3' thymine and +3 adenine) compared to the Z/Nap ZRE1 structure, as well as the different sidechain conformations of Asn182'. (C) Z Cys189 forms van der Waals contacts with the −2' thymine unique to Rp ZRE3. Van der Waals contacts between the −2' thymine position unique to the Rp ZRE3 DNA molecule and Cys189 are highlighted (yellow contour). This interaction is greatly reduced with a guanine present at the −2' position (purple contour). Additionally, mutation of Cys189 to a serine residue greatly reduces the van der Waals interaction (red contour) with the −2' Rp ZRE3 thymine.

To explore why methylation of some ZRE sites is required for Z binding, we used the Sybyl program to model the interaction of Z with the newly discovered methylated Nap ZREs versus the AP1 site. Consistent with previous observations modeling the binding of Z to methylated ZREs in the Rp [35],[46], our model of Z binding to the methylated Nap ZRE2 left-half site (Fig. 10A, upper panel) indicates that the cytosine methyl group at +1' occupies the same position as the thymine methyl group (+1') in the AP1 site (Fig. 10A, lower panel). Interestingly, this cytosine is present in the same location within the left half-sites of all four methylation-sensitive ZREs (Fig. 9).

We also observed that methylation of cytosines in the Nap ZRE2 site helps to define the sidechain conformation of the Z Ser186 and Asn182 residues, and stabilize a hydrogen bonding network. Upon methylation, the two cytosine methyl groups (+1' and −2) limit the sidechain movement of Ser186, stabilizing its hydrogen bonds to the amine groups of both cytosines (+1' and −2) and to the carbonyl group of Asn182. This helps define the sidechain of Asn182 to a different conformation which allows it to form hydrogen bonds with several DNA bases, including the guanine at +2' which is present in the left half-site of all four methylation-sensitive ZREs (Fig. 9). In addition, this conformation of Asn182 stabilizes a hydrogen bond with the adenine at −3 in Nap ZRE2 (Fig. 10A). The adenine at −3, which is occupied by a thymine in the other methylated and unmethylated ZREs, as well as AP1 (Fig. 9), is unique to the Nap ZRE2 site. Thus, the hydrogen bonds in this network appear to be most abundant with the Z/Nap ZRE2 complex, helping to explain why the Nap ZRE2 interaction with Z absolutely relies on methylation. In addition, this architecture explains why mutation of Ser186 to alanine, which would attenuate this hydrogen bonding network, greatly abrogates Z binding to methylated Nap ZRE2.

A model of Z bound to the right half-site of the methylated Nap ZRE1 site, which has a unique CpG not present in the other methylated ZREs, is shown in Fig. 10B. Similar to position +1', the methylated cytosine at position −3' of Nap ZRE1 mimics the thymine present at −3' in AP1 and all other methylated ZREs (Fig. 10B). Our model demonstrates that methylation of the cytosines in the right half-site of Nap ZRE1 helps to stabilize the sidechain of Asn182' to a different conformation, allowing it to form hydrogen bonds with the altered nucleotides at −3' and +3. Methylation of the cytosine at −3' also defines the sidechain conformation of Ser186' and Arg190', and stabilizes the hydrogen bonds between Ser186' and Arg190', and between Arg190' and two phosphate groups of DNA backbone.

Interestingly, the Rp ZRE3 right half-site has a unique thymine at −2' (Fig. 9), which leads to van der Waals contacts of this thymine to Cys189 (Fig. 10C, yellow contour). This interaction is absent in other ZREs and AP1, which have a common guanine at this position (Fig. 10C, purple contour). Mutation of Cys189 to a serine residue (Fig. 10C, red contour) is predicted to greatly reduce the van der Waals contact to the −2' thymine of Rp ZRE3. Collectively, this explains why Cys189 is most important for Z binding to Rp ZRE3.

## Discussion

The EBV Z protein, which mediates the switch from latent to lytic viral infection, is to date the only transcription factor shown to preferentially bind to, and activate, the methylated form of a target promoter. However, only one EBV promoter (Rp) has previously been shown to be preferentially bound, and activated, by Z in the methylated form. Indeed, all other previously identified Z binding sites in early lytic promoters do not even contain CpG motifs. Thus, whether methylated CpG-containing ZRE sites are specifically important for Z activation of the Rp, but not other viral promoters, has remained unclear. In this report, we identify two CpG-containing ZREs in the EBV early lytic Nap, and show that these ZREs are only bound by Z in the methylated state. Furthermore, we demonstrate that the Nap can only be activated by Z efficiently *in vivo* when the promoter is methylated. In addition, we show that both Nap ZREs are generally methylated in the EBV genome of latently infected cells. Our molecular modeling studies of Z bound to these new Nap ZREs help to explain why methylation of these sites is required for Z binding. Together, these results indicate that methylation of the EBV genome not only enhances Z activation of R transcription, but is required for Z activation of Na transcription. This apparently unique feature of Z likely helps the virus to efficiently activate lytic viral gene transcription when the viral genome is highly methylated. Conversely, the inability of Z to induce expression of the viral transcription factors, R and Na, from the incoming (unmethylated) viral genome may help the virus to establish latency.

Our laboratory previously showed that Z preferentially binds to and activates the methylated EBV *BRLF1* gene promoter, Rp, through two CpG-containing ZREs. Z does not bind to all methylated CpG motifs, but rather binds to methylated CpGs embedded within ZRE-like motifs [22],[34]. In the case of the Rp, we found that Z binds to both the unmethylated, as well as methylated, forms of the Rp ZRE2 and Rp ZRE3 sites (Fig. 4A) (although binding to the methylated form is much more efficient, particularly in the case of the Rp ZRE3). The Rp also has one ZRE (Rp ZRE1) which does not contain a CpG and therefore cannot be methylated. Of note, while Z activates the methylated form of the Rp more efficiently than the unmethylated form, it clearly also activates the unmethylated form of Rp (Fig. 3A). The ability of Z to activate unmethylated Rp is likely mediated by binding to the unmethylated Rp ZRE1 and ZRE2 sites.

In the case of the Nap, both of the ZREs identified contain CpG motifs, and require methylation for Z binding. Furthermore, Z binding to the highly atypical Nap ZRE1 site (T**C**GCC**C**G), which contains two CpG motifs, requires methylation of both CpGs. Consistent with this methylation-dependent binding of Z to the Nap *in vitro*, we found that methylation of the Nap is required for efficient Z activation of a Nap driven luciferase reporter construct *in vivo*. In fact, Z activation of the Nap may be even more dependent upon promoter DNA methylation than Z activation of Rp. Site-directed mutation of either ZRE in the Nap greatly abrogated Z activation of the methylated Nap-luciferase construct, indicating that the presence of both ZREs is important for Z activation. While Nap methylation is required for Z activation, we found that it has the opposite effect on the ability of cellular transcription factors to activate the Nap. Thus, although the Nap has high constitutive activity in the unmethylated form, it is completely inactive in the methylated form unless Z is present.

Although a previous report suggested that bacterially produced His-tagged Z protein can bind to the unmethylated form of Nap [18], we found only weak Z binding to the unmethylated form of the Nap using *in vitro* translated Z as a source of protein in EMSAs. Therefore, a post-translational modification of Z that does not occur in bacterial cells, or an interaction with another cellular protein(s), may be required to observe methylation-dependent Z binding to the Nap ZREs. Z was also reported to activate the unmethylated form of Nap in a reporter gene assay [18]. We speculate that this previous result may reflect the ability of Z to activate certain reporter gene constructs through a non-DNA binding mechanism when expressed at very high levels and/or the presence of cryptic AP1 sites in some reporter gene vectors [47]. To date, Z activation of lytic viral genes in the context of the intact viral genome requires direct Z binding to the viral promoters. Cellular factors could potentially stabilize weak Z binding to the unmethylated form of Nap in some cell types.

We and others have previously shown that a Z(S186A) mutant binds very poorly to the methylated forms of Rp ZRE2 and Rp ZRE3, even though this alteration does not affect Z binding to the consensus (and unmethylated) AP1 motif [22]. Furthermore, the Z(S186A) mutant cannot disrupt viral latency when transfected into latently infected cells, and this phenotype is at least partially reversed when the R protein is supplied in trans, expressed under the control of a constitutively active promoter [36],[37]. In this paper, we have shown that the Z mutant, Z(S186A), is severely impaired for binding to each of the four known methylated ZRE sites in EBV. Consistent with its methylation-dependent binding defect, the Z(S186A) mutant is unable to activate the methylated Nap reporter construct *in vivo*, or to induce Na expression from the methylated viral genome in latently infected cells even in the presence of a co-transfected R expression vector. The finding that the Z(S186A) mutant is defective not only for *BRLF1* (R) gene activation, but also for *BRRF1* (Na) gene activation, suggests that loss of both R and Na protein expression may contribute to its phenotype.

Although the crystal structure of Z bound to the consensus AP1 site has been published [35], the crystal structure of Z bound to a methylated ZRE site has not yet been reported. Karlsson *et al.* recently suggested that Z residue Ser186 stabilizes Z binding to the left half-sites of methylated Rp ZRE2 and Rp ZRE3 by directly interacting with the methyl group of the cytosine located at position +1', and thereby stabilizing a hydrogen bonding network involving Ser186, Asn182 and the guanine located at +2' [46]. Our models agree with this notion except that more hydrogen bonds were identified in this network, particularly in Z bound to Nap ZRE2 (Fig. 10A, upper panel). The difference in the hydrogen bond network of Z/Nap ZRE2 appears to be contributed by its unique adenine at the −3 position. Its hydrogen bond to Asn182 requires that the carbonyl and the amine groups of the Asn182 sidechain are in the opposite direction (Fig. 10A, upper panel) compared to AP1 (Fig. 10A, lower panel). We hypothesize that stabilization of this conformation in Nap ZRE2 relies more on this methylated CpG motif than in Rp ZRE2, which differs from Nap ZRE2 only at the −3 position. This is consistent with our observation that unmethylated Nap ZRE2 fails to bind Z while unmethylated Rp ZRE2 can still bind. In all of our models, Ser186 appears to play a central role in bridging the hydrogen bond networks, consistent with the observation that Z(S186A) is impaired for binding to all four known methylated ZREs in EBV. In addition to the left half-site, Ser186' clearly plays a role in forming a second hydrogen bond network, interacting with the unique CpG motif on the right half-site of Nap ZRE1; this network also relies on methylation of the CpG motif (Fig. 10B). The sidechain conformation of Asn182' likewise changes in context of the altered sequence of the Nap ZRE1 right half-site for hydrogen bond formation (Fig. 10B). Thus, the ability of Z to recognize different ZREs partially relies on stabilization of Asn182 conformations for formation of hydrogen bonds that vary depending on the context of different DNA sequences.

Consistent with previous observations, the common CpG motif at the left half-site gives rise to a methylated cytosine at the +1' position in all four methylation-sensitive ZREs that mimics the structure of thymine in AP1 (Fig. 10A). Interestingly, the unique CpG motif at the right half-site of Nap ZRE1 also gives rise to a methylated cytosine at the −3' position that mimics the structure of the common −3' thymine present in most other ZREs, as well as in AP1 (Fig. 10B). This observation suggests that the ability of methylated cytosines to mimic thymine in DNA-protein interactions might be a more common phenomenon.

Another factor regulating the binding of Z to DNA is the redox state of the bZIP domain [48],[49]. This regulation is conserved in many of the bZIP proteins, including c-fos and c-jun. In contrast, DNA binding of C/EBPα, which contains a serine residue in the place of the usual cysteine residue, is insensitive to the redox environment. Z possesses the bZIP protein conserved cysteine at residue 189, and the DNA-binding function of Z is redox dependent [49]. Karlsson *et al.* recently reported that Z(C189S) is defective in binding to the methylated Rp ZRE3 site (although this defect was not redox dependent), and based on this finding proposed that Z Cys189 directly contributes to Z binding to at least some methylated ZREs [46]. In our experiments, we confirmed that Z(C189S) is highly impaired for binding to the methylated form of the Rp ZRE3 site, as well as the Nap ZRE1 site, but much less impaired for binding to the methylated forms of Nap ZRE2 and Rp ZRE2. These results suggest that Z Cys189, in contrast to Z Ser186, is required for binding to a limited subset of methylated ZREs. Consistent with the previous model of Z bound to Rp ZRE3 [46], our model likewise predicts that van der Waals contacts occur between the −2' thymine position unique to the Rp ZRE3 DNA molecule and Z Cys189 (Fig. 10C). However, the Z(C189S) mutant is not predicted to have these interactions. Our finding that Z(C189S) binds to some methylation-dependent ZREs more efficiently than to the Rp ZRE3 site may reflect the absence of a thymine at the −2' position in these other ZREs. As previously reported [46], we found that the Z(C189S) mutant induces lytic infection in latently infected 293 cells almost as well as wild-type Z, perhaps reflecting our previous finding that some Rp ZREs are unmethylated in this cell type [22].

The fact that the Rp and Nap are potentially the only EBV promoters which are preferentially bound, and activated, by Z in the methylated form suggests that these two promoters may be activated in tandem. Since our laboratory has previously shown that Na cooperates with R to activate the Z promoter in the context of the intact viral genome [19], the ability of the Z protein to activate both R and Na transcription efficiently from the methylated form of the viral genome may serve as an essential positive autoregulatory loop in the initial stage of viral reactivation. However, since the EMSA assays performed to search for ZREs in lytic EBV promoters have not generally used methylated DNA probes, many undiscovered methylation-dependent ZREs may yet exist in the EBV genome. Whether there are also methylation-dependent ZREs in the cellular genome remains unknown.

We previously showed that demethylation of the EBV genome in latently infected 293 cells inhibits the ability of Z to induce lytic viral gene expression [34], strongly suggesting that viral genome methylation promotes the ability of Z to activate lytic genes in the context of the intact viral genome. Based upon the results presented here, we propose the following model to illustrate how EBV uses R and Na promoter methylation to enhance both the latent, and lytic, forms of infection. In cell types (such as oropharyngeal epithelial cells) where viral infection is normally completely lytic, and thus the viral genome does not become methylated, we propose that R and Na, rather than Z, may be the primary mediators of lytic gene expression, since the ability of R and Na to induce Z expression does not require viral genome methylation (and in fact may be inhibited by methylation). During infection of cells (such as B cells) that normally undergo the latent form of infection, the inability of Z to activate R and Na transcription efficiently from the incoming unmethylated viral genome helps the virus to establish latency. Following establishment of viral latency, the viral genome eventually becomes highly methylated (potentially reflecting the ability of the virally encoded latent LMP1 protein to activate expression of the cellular methyltransferase 1 protein [50],[51]). Methylation of the Na and R promoters inhibits their ability to be activated by cellular transcription factors, further promoting viral latency as long as the Z protein is not available. However, following reactivation of Z expression by stimuli such as plasma cell differentiation, methylation of the Na and R promoters enhances Z binding, perhaps ensuring that a limiting amount of Z preferentially binds to ZREs in the R and Na promoters. Alternatively, the conformation of full-length Z bound to methylated ZREs may be subtlety different from its conformation bound to unmethylated ZREs. If so, Z bound to methylated ZREs may interact more strongly with co-activator proteins such as CBP, and thus activate promoters with methylated ZREs more efficiently [52]. In combination, expression of the Z, R and Na proteins is then sufficient to initiate the entire lytic cascade of viral gene expression.
